# Bacterial-mediated RNAi and functional analysis of *Natalisin* in a moth

**DOI:** 10.1038/s41598-021-84104-0

**Published:** 2021-02-25

**Authors:** Xia-Fei Wang, Zhe Chen, Xu-Bo Wang, Jin Xu, Peng Chen, Hui Ye

**Affiliations:** 1 Yunnan Academy of Biodiversity, Southwest Forestry University, Kunming, 650224 China; 2 School of Life Sciences, Yunnan University, Kunming, 650091 China; 3 Yunnan Academy of Forestry and Grassland, Kunming, 650201 China

**Keywords:** Biological techniques, Molecular biology, Zoology

## Abstract

The neuropeptide natalisin (NTL) has been determined to play essential roles in reproduction in two Diptera and one Coleoptera species. Whether NTL has similar or even different functions in Lepidoptera remains to be determined. Here, we cloned the NTL transcript in the common cutworm moth *Spodoptera litura*. This transcript encodes a 438-amino acid protein. Twelve putative *Sl*-NTL neuropeptides were defined by cleavage sites. These NTL peptides share a DDPFWxxRamide C-terminal motif. The expressions of *Sl*-NTL is low during the egg and larval stages, which increased to a higher level during the pupal stage, and then reached the maximum during the adult stage. Moreover, the expression pattern during the pupal stage is similar between sexes while during the adult stage, it is dimorphic. To explore the function of *Sl*-NTL and assess its potential as a target for pest control, we knocked down the expression of *Sl*-NTL in both sexes by using bacteria-mediated RNAi. This technique significantly down regulated (reduced up to 83%) the expression of *Sl*-NTL in both sexes. Knocking down *Sl*-NTL expression did not significantly affect its development, survival and morphology but significantly reduced adults’ reproductive behavior (including female calling, male courtship, mating and remating patterns and rates) and reproductive output (offspring gain reduced more than 70%).

## Introduction

Insects are currently the most speciose animal group, including nearly a million of species that occur almost everywhere on the earth^1,2^. The study of insect physiological and behavioral adaptations and diversity not only will facilitate our understanding of sexual selection mechanisms and evolutionary ecology of insects, but also will be helpful for the development of novel strategies to control pest species with a reduced or minimal harm to the environment and especially to non-target species^3–5^. Neuropeptides are one of the key players in information transfer, acting as important regulators of physiology and behavior in relation to development and reproduction in insects.

Tachykinin-related peptides (TRP)^6^ and Natalisin (NTL)^7^ are closely related neuropeptides found in insects. TRP and NTL share the common sequence motif FxxxRamide (C-terminally amidated), which is FxGxRamide in TRPs while in NTL is FxPxRamide for Diptera and FWxxRamide for Coleoptera and other insects. Similarly, the receptors of TRP and NTL are also closely related but unequivocally form separate clusters^7,8^. All known TKRP receptors are class A G-protein coupled receptors (GPCRs)^6,9^. One of these GPCRs, which was previously known as TRP receptor (TRPR)^10,11^, was identified as the NTL receptor (NTLR)^7^. Tachykinin-related peptides have multiple functions in insects, such as myotropic activity^12^, diuretic function^13^, control of lipid metabolism^14^ and modulation of olfactory neurons^15,16^. Comparatively, the function of NTL is exclusively linked to reproduction of insects. Knockdown of NTL by transgenic RNAi in *Drosophila melanogaster* showed lower mating rate and shorter inoculation duration than those of wild-type flies, whereas knockdown of NTL by dsRNA injection in *Tribolium castaneum* resulted in lower egg production than controls^7^. In the oriental fruit fly, *Bactrocera dorsalis*, RNA interference mediated by dsRNA injection in adults significantly reduced male and female mating frequencies^8^. Furthermore, a study showed that tachykinin controlled male-specific aggressive behaviour in *D. melanogaster* by acting on the NTLR^17^, suggesting that the NTL and tachykinin signalling systems might interact with each other.

So far, NTL orthologs have been found in almost twenty insect species from five orders, such as *D. melanogaster*^7^ and *Glossina morsitans morsitans*^18^ from Diptera, *T. castaneum*^7^ and *Nicrophorus vespilloides*^19^ from Coleoptera, *Bombyx mori*^7^ and *S. exigua*^20^ from Lepidoptera, and *Locusta migratoria*^21^ from Orthoptera. NTL orthologs have also been found in non-insect organisms, such as mites^22,23^ and crayfish^24^. However, the function of NTL has only been determined in three species from two insect orders, including *T. castaneum* from Coleoptera, *B. dorsalis* and *D. melanogaster* from Diptera^7,8^. Whether NTL has similar or even different functions in other insect or non-insect species remains to be determined. Interestingly, Jiang et al.^22^ found that the honey bees *Apis mellifera* lack both NTL and the NTL receptor in their genome sequences, while its ectoparasite *Varroa destructor* have these two genes, providing a foundation for the development of novel varroa-mite-specific control agents.

In the present study, we identified the NTL transcript in the common cutworm moth *Spodoptera litura* and characterized its structure and phylogenetic status among insect and non-insect species. We then tested the mRNA expression pattern of NTL in relation to different developmental stages and tissues of males and females. To shed some light on the function of NTL and explore its potential as a target for novel insecticides, we knockdown NTL expression in both sexes by using bacteria-mediated RNAi and tested whether and how silencing of NTL will affect the developmental and reproductive fitness in this insect. To our knowledge, this is the first study to investigate the function of NTL in Lepidoptera and for the first time using bacteria-mediated RNAi in *S. litura*.

RNA interference (RNAi) is a valuable tool for gene functional analysis and determination, and also is an emerging technology to provide novel approaches for pest control in agriculture and disease treatment in humans^25^. In insects, RNAi can be induced by injection of in vitro synthesized dsRNA or the oral route, either by feeding synthesized dsRNA directly or by feeding bacteria expressing the dsRNAs in vivo (bacteria-mediated RNAi)^26,27^. Bacterially expressed dsRNA is cheaper than producing dsRNA in vitro with a kit, particularly when used in large scale gene function analysis^28^. More importantly, bacteria-mediated RNAi is a promising technology to provide sustainable and environmentally sound approaches to control insect pests and plant pathogens^25^.

*Spodoptera litura* Fabricius (Lepidoptera: Noctuidae) is known as tobacco cutworm or cotton leafworm, and is one of the world’s key agricultural pests due to its strong pesticide resistance, alternating generations and omnivorous characteristics. The extensive and indiscriminate use of pesticides for the prevention and control of this pest have caused severe damage to the environment and even further increased its insecticide resistance^29–31^. Therefore, sustainable and environmentally sound control strategies such as bacteria-mediated RNAi based control techniques^32–35^ are imperative to control this pest.

## Methods

### Insects

*Spodoptera litura* larvae were reared on an artificial diet^36^ at 25 ± 1 °C and a relative humidity of 60–70% with a photoperiod of 14:10 h light:dark photoperiod regime. Newly eclosed male and female moths were maintained in separate cages to ensure virginity. Adult moths were maintained in the same environment and fed with 10% honey solution.

### Molecular cloning

Total RNA was extracted from adult moths with Trizol (Takara, China) according to the manufacturer’s protocol, and the purity and concentration of RNA was measured by using a spectrophotometer (NanoDrop 2000, USA).

We obtained a partial mRNA fragment (3′ end missing) of *NTL* from *S. litura* in our previous RNA-seq analysis. Based on the partial sequence, gene specific primer (GSP) and nested gene specific primer (NGSP) were designed and synthesized for 3′-RACE (Table 1)*.* The first round of 3′-RACE amplification was performed by using 3′-ready-cDNA with UPM and GSPf (SMARTer Kit, Clontech, USA); then the first round product was diluted at 1:100 and used as templates for nested PCR reactions with UPM and GSPf. All amplifications were performed with 50 μl reaction mixtures containing 1 μl of template, using the same program: 94 °C for 5 min, followed by 5 cycles of 94 °C for 30 s, 72 °C for 4 min, and 5 cycles of 94 °C for 30 s, 70 °C 30 s, 72 °C 4 min; then 25 cycles of 94 °C for 30 s, 68 °C 30 s, 72 °C for 4 min; and a final extension step of 72 °C for 10 min. The PCR products were then cloned into trans1-T1 cells using pEASY-T5 zero vector system (TransGen, China). The plasmids were isolated using TIANPrep (TransGen, China) and were sequenced by Beijing Huada Biological Company.

**Table 1:** Primers for RACE, qPCR and RNAi.

Primer names	Primer sequences (5ʹ–3ʹ)	Uses
NTL-GSPr	GAGGCAGGAGAGAAACAGACGACCCTTT	3′-RACE
NTL-NGSPr	GGGTAACCGTGGAAGGCGGAAGAC	3′-RACE
NTL-Qf	AGACAGGCGTGGTGCTATTG	qPCR
NTL-Qr	TTCCTCTTTGTGGAGTGTAGTTCC	qPCR
Actin-Qf	CATCTACGAAGGTTACGCCCT	qPCR
Actin-Qr	AGCGGTGGTGGTGAAAGAGTA	qPCR
dsNTL-F	AAGGAAAAAA**GCGGCCGC**GCGTGGTGCTATTGAAGA	RNAi
dsNTL-R	CCG**CTCGAG**TGTAATTGAGCTGCCAGTT	RNAi
dsEGFP-F	AAGGAAAAAA**GCGGCCGC**AAGCAGCACGACTTCTTC	RNAi
dsEGFP-R	CCG**CTCGAG**GCTCAGGTAGTGGTTGTC	RNAi

Underlined were protecting nucleotides and bold ones were *Not*I and *Xho*I restriction sites.

### Gene expression in relation to development, sex and tissues

The development duration of *S. litura* eggs, larvae and pupae is about 3, 18 and 11 days, respectively^37^. Adult moths can live up to 10 days but most matings and ovipositions occurred in the first three days after eclosion^37^. Therefore, in the present study, we collected samples from 1-d-old eggs (10 eggs per sample), 0- (1st instar), 9- (3rd instar) and 18-d-old (4th instar) larvae, 0-, 6- and 11-d-old male and female pupae, and 0-, 1- and 2-d-old adult male and female virgin moths (5 insects per sample), respectively. Heads, thoraxes and reproductive systems were also sampled from 0-d-old male and female adults (8 insects per sample). Sampled female reproductive systems included oviduct, vestibulum, bursa copulatrix, ductus seminalis, spermatheca and its duct and gland, while male reproductive systems contain vesicula seminalis, vas deferens, accessory glands and ductus ejaculatorius^38^. Total RNA was extracted from each of these samples using a RNA prep pure Tissue kit (TianGen, China). The purity and concentration of the RNA were assessed as above. Three replicates were used for each category.

First strand cDNA synthesis was performed using a PrimeScript RT reagent Kit (Perfect Real Time) (TaKaRa, China). Real-Time PCR was performed with gene specific primers for *Sl-NTL* (Table 1) using the TB Green Premix Ex Taq II (TaKaRa, China) in a volume of 25 μl. *Actin* was used as a reference gene^39,40^. Reactions were run in triplicate on the QuantStudio 7 Flex (Thermo Fisher Scientific, USA) using the following reaction condition: 95 °C for 5 min followed by 40 cycles of 95 °C for 30 s, 60 °C for 34 s. Analysis of the dissociation curves for the target and reference genes showed a single melt peak and the efficiencies of the target and reference genes were similar. The 2^−ΔΔ^^CT^ method^41^ was used to calculate the relative quantities of the target genes.

### Vector construction and dsRNA preparation

A 255 bp fragment (nucleotide 1055–1309 of *Sl-NTL* transcript, GenBank ID: MK673156) was designed for RNAi target by using siDirect version 2.0. This fragment was amplified by RT-PCR using total RNA as a template and primers (Table 1) containing *Not*I and *Xho*I restriction sites. Amplification reactions comprised 30 cycles of 94 °C for 30 s, 60 °C for 30 s and 72 °C for 60 s, with a final extension step of 72 °C for 10 min. PCR products were confirmed by using a 1.5% agarose gel and purified by using a SanPrep DNA Gel Extraction Kit (Sangon Biotech, China). The PCR product was then cloned into the plasmid L4440 (obtained from Addgene)^26,27^ between the *Not*I and *Xho*I sites. The resulting recombinant vector L4440-*Sl*NTL was introduced into competent HT115(DE3) cells. To produce dsRNA, single colonies of HT115(DE3) bacteria containing L4440-*Sl*NTL or L4440 vector were grown for 14 h with shaking in LB with 100 μg/ml ampicillin and 12.5 μg/ml tetracycline at 37 °C. Synthesis of T7 polymerase was induced by addition of IPTG to 0.4 mM and the bacteria were incubated under shaking for an additional 4 h at 37 °C. The expressed NTL dsRNA was extracted from the bacteria by using RNAiso Plus (Takara, China) according to the manufacturer’s protocol. The length of the NTL dsRNA was confirmed by electrophoresis on 1% agarose gel. The quantity of NTL dsRNA was estimated by comparing the brightness of the NTL dsRNA band and a quantified RNA maker. The production of NTL dsRNA under above bacteria incubation system is about 7–9 μg dsRNA/1 ml bacteria culture.

A 372 bp fragment of the enhanced green fluorescence protein (EGFP) gene was selected as a control dsRNA and was amplified using designed specific primers (Table 1). The recombinant plasmid for EGFP dsRNA expression protocol was the same as that for L4440-*Sl*NTL. The production of GFP dsRNA was also confirmed and quantified as above. The production of GFP dsRNA is also about 7–9 μg dsRNA/1 ml bacteria culture.

### Bacterial-mediated RNAi and silencing efficiency

**Figure 1:**
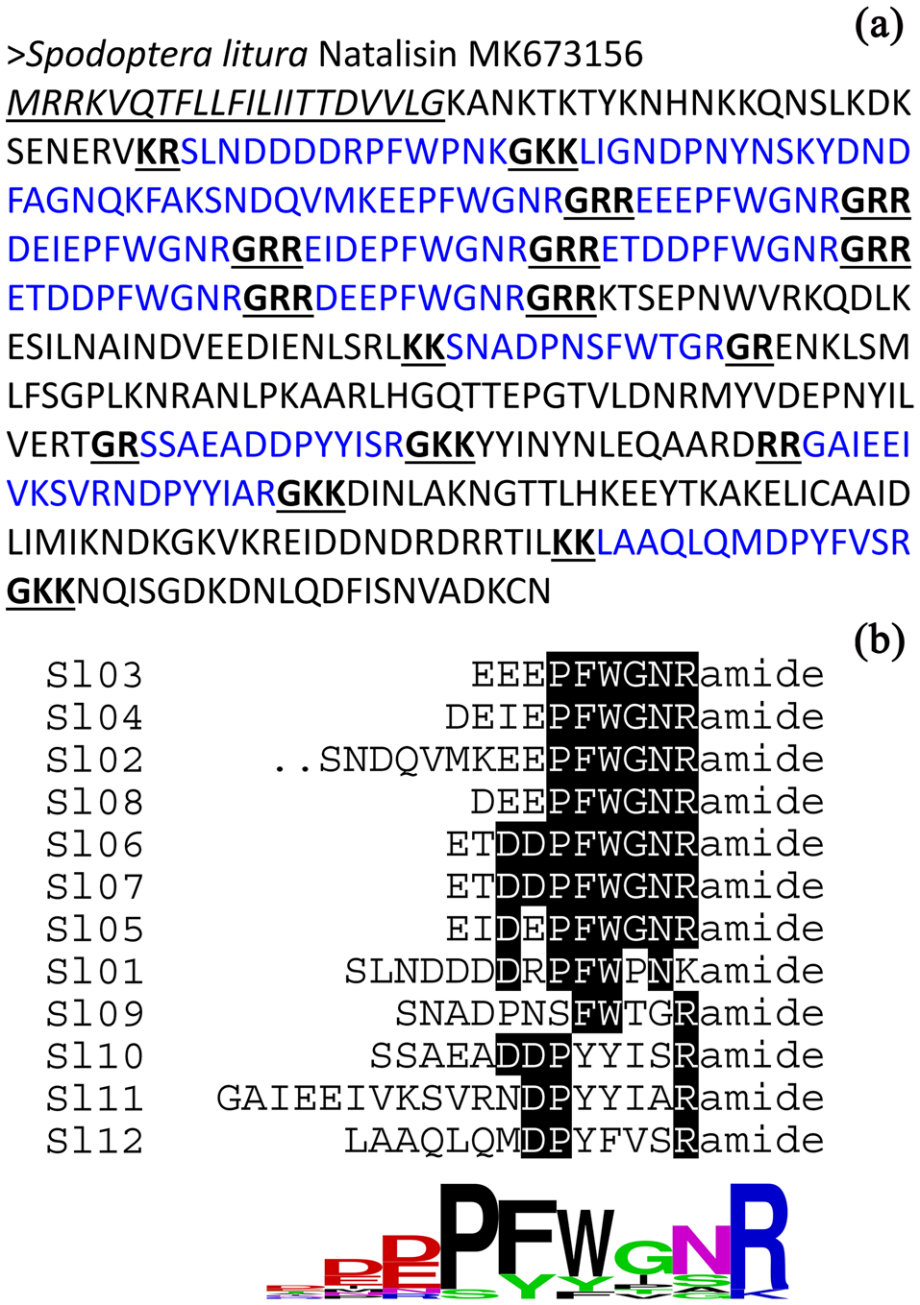
Deduced amino acid sequences of NTL precursors (**a**) and prediction of mature peptides (**b**). The putative signal peptide at the N terminus is in italics and underlined. Canonical amidation with di-basic signals is marked by bold and underlined fonts. The putative mature peptides were marked by blue fonts. The calculated consensus logo is shown at the bottom.

Above confirmed bacteria cultures were used for RNAi. To prepare bacterial cells for feeding, bacterial cells were collected from 30 ml IPTG-induced culture by centrifugation at 10,000 g for 2 min, resuspended in 1 ml sterile water. During our preliminary experiments, we have tried one-time ingestion test^25^ by feeding the third instar larvae with 20 μl per larva of the prepared bacteria solution, i.e., feed the solution only once in the lifetime. However, qPCR test of the expression of *Sl-NTL* in treated mature larvae and adults showed a low silencing efficiency (< 30%). We thus selected to use the continual ingestion method^25^ that may have better silencing efficiency in the present study. Briefly, the third instar larvae (9-d-old) were individually caged in 4 cm × 4 cm × 4 cm plastic cells and were fed with 20 μl of the prepared bacteria solution (dropped on the surface of the food) each day until larvae mature (18-d-old, stop feeding and preparing for pupation). *S. litura* larvae are gluttonous and thus starvation before bacteria feeding (often used in other insect species)^25^ is unneeded. To confirm the silencing efficiency, bacteria fed mature larvae (18-d-old) and 0-d-old males and 1-d-old females (maximum NTL expression during adult stage, Fig. 1) were collected and the transcription levels of *Sl-NTL* were quantified by qPCR as above. The heads, thoraxes and reproductive systems of adults were also dissected as above. Larvae feeding on the food added with 20 μl distilled water and food added with 20 μl solution containing the bacteria expressing EGFP dsRNA were set as controls. Three replicates were used for each treatment.

### Effect of NTL silencing on male and female reproductive fitness

The third instar larvae were individually fed with bacteria expressing *Sl-*NTL dsRNA continuously as above. Larvae feeding on the food added with distilled water and bacteria expressing EGFP dsRNA were also set as controls as above. After pupation, the male and female pupae were sexed according to the morphology of exterior paramera^36^ and maintained in separate cages to ensure virginity of adults after eclosion. Eclosed female and male moths were collected and reared in separate cages by feeding with 10% honey solution until they were used for the following experiments. Developmental duration, morphology and survival were also observed and recorded.

To determine the effect of NTL silencing on male reproductive behavior, L4440-*Sl*NTL treated and control virgin males (n = 13) were paired with wild virgin females (one pair per box) at the beginning of the second scotophase after eclosion. Male reproductive behaviors were observed in the following two scotophases after pairing. Similarly, to determine the effect of *NTL* silencing on female reproductive behavior, L4440-*Sl*NTL treated and control virgin females (n = 13) were paired with wild virgin males (one pair per box) at the beginning of the second scotophase after eclosion. Female reproductive behaviors were observed in the following two scotophases after pairing.

Following above treatments, behaviors were observed every 10 min by quickly scanning all pairs and recording the following: male courtship—the male jumping and fanning his wings over or around the female or if the male exposed his genitalia trying to engage the female’s genitalia; female calling—the area of the female ovipositor bearing the pheromone gland is extruded and exposed to the outside^42^; mating—the two insects engaged by the tip of the abdomen. The events and duration of female calling (a calling can be short, a few minutes, or long, a few hours, and usually can last up to tens of minutes) and mating behaviors (the mating duration of this species is about 45 min)^43^ were recorded. Male courtship is instantaneous (usually lasts a few seconds to tens of seconds) and thus is hard to record its duration. We thus recorded the number of courtships but did not record the duration of each courtship. The moths were provided with honey solution as food and paper strips as oviposition substrates. Illumination during observation was provided by a 15 W red light. Their lifetime mating patterns were recorded by observing all paired insect half-hourly during the scotophase as the mating duration of this species is about 45 min^43^. The first two days’ and lifetime fecundity (no. of eggs laid) and longevity were recorded. Matings were verified by dissecting dead females to count the number of spermatophores in their bursa copulatrix.

*S. litura* eggs are laid in clusters and are usually several layers thick (mostly 2–3 layers). Eggs adhered to each other and the paper tightly, and thus trying to separate them to count the numbers are very likely to damage the eggs. Therefore, we cut the egg clusters with the paper and then it was weighed (Wt1). The eggs were then incubated in boxes that contained food. After larvae emergence, the clean paper was weighed again (Wt2). The number of eggs was calculated by (Wt1–Wt2)/(weight of one egg). The weight of one egg was 0.046 mg, which was determined by weighing more than 10 thousand eggs and calculated previously. The number of emerged larvae were also recorded.

### Statistics

Data on gene expression, number of eggs (the first two days after pairing and lifetime) and longevity were analysed using an ANOVA followed by Fisher’s LSD test for multiple comparisons. Data on reproductive behavior (calling, courtship and mating) and number of larvae were not normally distributed even after transformation and thus were analysed using the nonparametric Kruskal–Wallis test followed by Dunn’s procedure for multiple comparisons^44^. All analyses were conducted using SPSS 20.0. The rejection level was set at *α* < 0.05. All values were reported as mean ± SE.

## Results

### Molecular cloning and phylogenetic analysis

Based on RNA-seq and RACE, the full-length mRNA sequence of the putative *NTL* (*Sl*-*NTL*; 1593 bp; GenBank accession: MK673156) was cloned from *S. litura*. This mRNA contained a putative ORF of 1317 nucleotides encoding a 438-amino acid protein (Fig. 1a), flanked by a 5′-UTR of 95 bp and a 3′-UTR of 181 bp. BLASTP analysis showed that *Sl*-NTL has high identity to NTLs from other lepidopterans, such as *S. exigua* (AXY04276.1; 94.92% identity), but lower identity to NTLs from species of other insect orders. The first 22 amino acid residues of the precursor were predicted as a signal peptide for secretion by using SignalP 4.0. Twelve putative *S. litura* NTL peptides were defined by flanking dibasic cleavage sites (combinations of K and R) and the canonical amidation site (G) at the C-terminus. Alignment of these putative NTL peptides showed a consensus sequence DDPFWxxRamide (‘x’ indicates a variable residue and ‘amide’ represents the amidated C-terminus) at the C-terminus (Fig. 1b).

**Figure 2:**
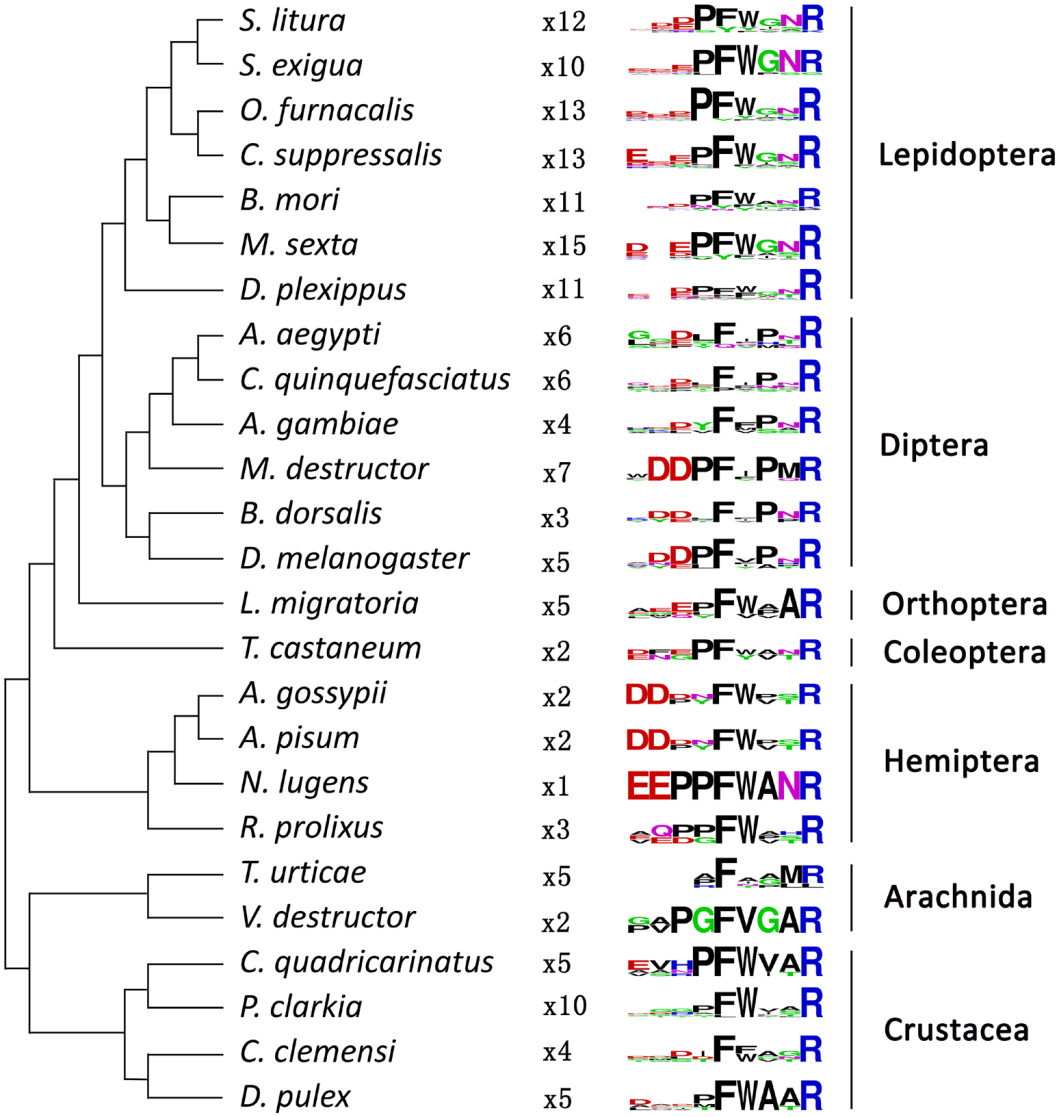
The species tree and C-terminal motifs of NTL (drawn based on Jiang et al.^7^). The tree is based on the species tree. The calculated consensus logos of the C-terminal motifs of NTL from different species were shown behind the species names. The numbers of the paracopies carrying the motif are shown by x followed numbers. NTL sequences were pooled and provided in Dataset S1 (supplemental file).

A hypothetical evolutionary tree and the C-terminal motifs of NTL from different organisms was drawn and presented in Fig. 2. The C-terminal motifs of NTL is FxPxRamide for Diptera while in other orders of Insecta, including Coleoptera, Lepidoptera, Orthoptera and Hemiptera, is FWxxRamide.

### Gene expression in relation to development, sex and tissues

**Figure 3:**
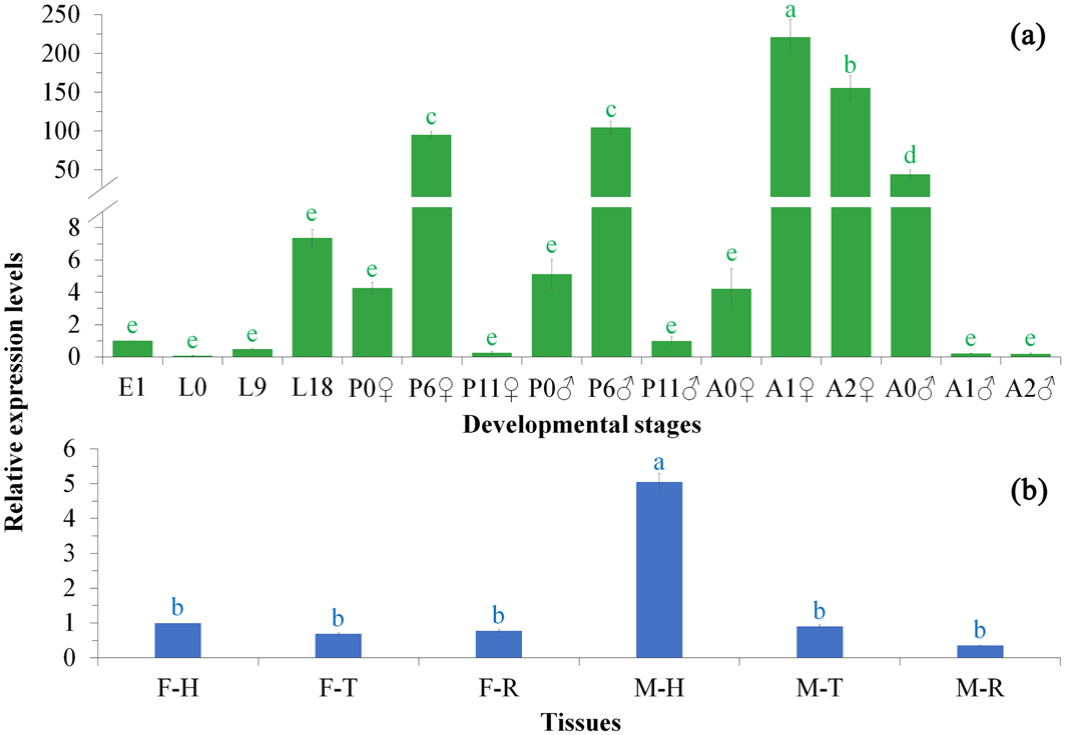
The expression of *Sl*-*NTL* in relation to development, sex and tissues. (**a**) expression patterns in the eggs and whole body of insects from different developmental stages. E1 refer to 1-d-old eggs; L0, L9 and L18 refer to 0-(1st instar), 9-(3rd instar) and 18-d-old (6th instar) larvae; P0♀, P6♀ and P11♀ refer to 0-, 6- and 11-d-old female pupae; P0♂, P6♂ and P11♂ refer to 0-, 6- and 11-d-old male pupae; A0♀, A1♀ and A2♀ refer to 0-, 1- and 2-d-old adult virgin females; and A0♂, A1♂ and A2♂ refer to 0-, 1- and 2-d-old adult virgin males. Expression in 1-d-old eggs was set as calibrator. (**b**) expression levels in 0-d-old male and female heads, thoraxes and reproductive systems. F–H, F–T and F–R refer to female heads, thoraxes and reproductive systems; M–H, M–T and M–R refer to male heads, thoraxes and reproductive systems; Expression in female heads was set as calibrator. For each parameter (Developmental stages or Tissues), bars with different letters are significantly different (*P* < 0.05); i.e., letters with the same color can be compared to derive the significance of difference between treatments.

Expression patterns of *Sl*-*NTL* in eggs and the whole body of insects from different developmental stages were examined using real time PCR and presented in Fig. 3a. *Sl*-*NTL* showed lower expression levels during the egg and larval stages, which increased to a higher level during the pupal stage, and then peaked during the adult stage. One-way ANOVA (*F*_15,32_ = 82.95, *P* < 0.0001) and post-hoc LSD test indicated that *Sl*-*NTL* showed the highest expression level in 1-d-old female adults (*P* < 0.05), followed by 2-d-old female adults (*P* < 0.05), then middle aged (6-d-old) male and female pupae (*P* < 0.05), and then 0-d-old male adults (*P* < 0.05). The expression in other stages were relative lower (*P* < 0.05).

Real-time fluorescence quantitative analysis also showed that *Sl*-*NTL* expressed in 0-d-old male and female heads, thoraxes and reproductive systems and showed significant variances (*F*_15,32_ = 54.45, *P* < 0.0001; Fig. 3b). The expression in male heads was significantly higher than that of thoraxes and reproductive systems (*P* < 0.05).

### Bacterial-mediated RNAi and silencing efficiency

In this study, we constructed the dsRNA expression vector by inserting *Sl*-*NTL* into the plasmid L4440 within the *Not*I and *Xho*I sites, and then the recombinant plasmid was transformed into competent HT115 (DE3) cells for dsRNA expression. At the same time, we used a non-related gene, *EGFP*, as a control for dsRNA expression.

To knockdown the expression of *Sl*-*NTL*, the third instar larvae were individually caged in plastic cells and were fed with 20 μl of the prepared bacteria solution each day until larvae mature. Relative expression levels of *Sl-NTL* in NTL dsRNA males and females (feeding *E. coli* expressing NTL dsRNA) reduced (reduction percentages ranged from 45.88% to 82.70%) significantly in comparison with controls (feeding sterile water or *E. coli* expressing EGFP dsRNA) (Table 2).

**Table 2:** Relative expression levels of the *S. litura NTL* in RNAi treated mature larvae and adults.

Sample	*NTL* relative expression levels					
	Water (control)^a^	GFP dsRNA (control)	NTL dsRNA	Reduction percentages^b^	*F*-value (ANOVA)	*P *value
Mature larvae (whole body)	1.00a	0.59 ± 0.28a	0.21 ± 0.09a	79.00; 64.41	*F*_2,6_ = 1.90	> 0.05
Female adults (whole body)	1.00a	1.22 ± 0.03a	0.37 ± 0.23b	63.00; 69.67	*F*_2,6_ = 36.16	< 0.001
Male adults (whole body)	1.00a	1.33 ± 0.69a	0.23 ± 0.33b	77.00; 82.70	*F*_2,6_ = 19.47	< 0.01
Heads of female adults	1.00a	1.05 ± 0.13a	0.36 ± 0.09b	64.00; 65.71	*F*_2,6_ = 17.02	< 0.01
Heads of male adults	1.00a	1.02 ± 0.20a	0.32 ± 0.06b	68.00; 68.03	*F*_2,6_ = 10.85	< 0.01
Thoraxes of female adults	1.00a	0.85 ± 0.13a	0.46 ± 0.12b	54.00; 45.88	*F*_2,6_ = 7.15	< 0.05
Thoraxes of male adults	1.00a	1.08 ± 0.09a	0.25 ± 0.05b	75.00; 76.85	*F*_2,6_ = 58.44	< 0.0001
Reproductive systems of female adults	1.00a	1.02 ± 0.16a	0.45 ± 0.14b	55.00; 55.80	*F*_2,6_ = 7.26	< 0.05
Reproductive systems of male adults	1.00a	1.02 ± 0.06a	0.23 ± 0.090.b	77.00; 77.45	*F*_2,6_ = 48.79	< 0.0001

^a^Water treated insects were used as calibrators. For each parameter (in each line), values with different letters are significantly different (*P* < 0.05).

^b^The first value is the Reduction Percentage relative to Water, and the second value is the Reduction Percentage relative to GFP dsRNA.

### Effects of NTL silencing on male and female reproductive fitness

Knock down NTL expression did not show significant effect on the development, survival and morphology in *S. litura*. The developmental duration and survival rate from the 3rd instar larvae (start to feed on bacteria) to adult emergence in treated and untreated insects were 19–21 days and 87–91%, respectively. No obvious morphological differences were found between treated and untreated insects.

**Figure 4:**
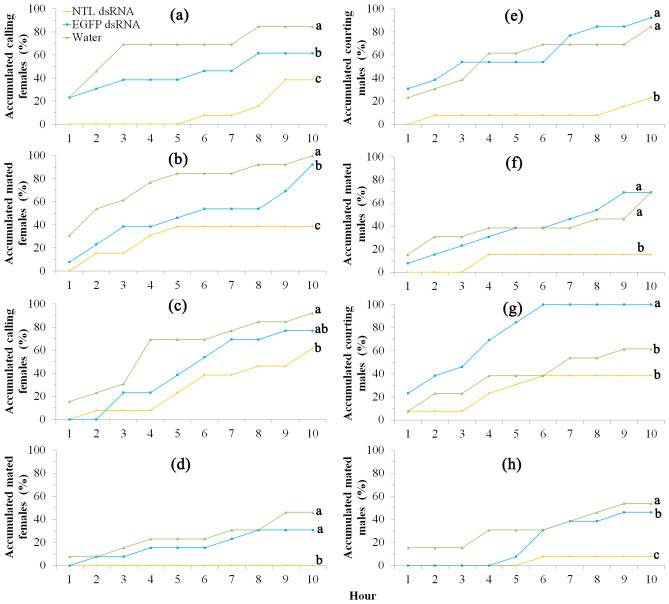
Effects of NTL RNAi on female (a-d; treated females paired with untreated males; thirteen pairs were used, i.e., n = 13) and male (e–h; treated males paired with untreated females; also thirteen pairs were used, i.e., n = 13) reproductive behavior in the first two days after pairing. Female calling behavior in the first (**a**) and second (**c**) scotophase. Female mating behavior in the first (**b**) and second (**d**) scotophase. Male courtship behavior in the first (**e**) and second (**g**) scotophase. Male mating behavior in the first (**f**) and second (**h**) scotophase. Data were not normally distributed even after transformation and thus were analysed using the nonparametric Kruskal–Wallis test followed by Dunn’s procedure for multiple comparisons. For each parameter, i.e., in each of the subgraphs, lines with different letters are significantly different (*P* < 0.05).

**Figure 5:**
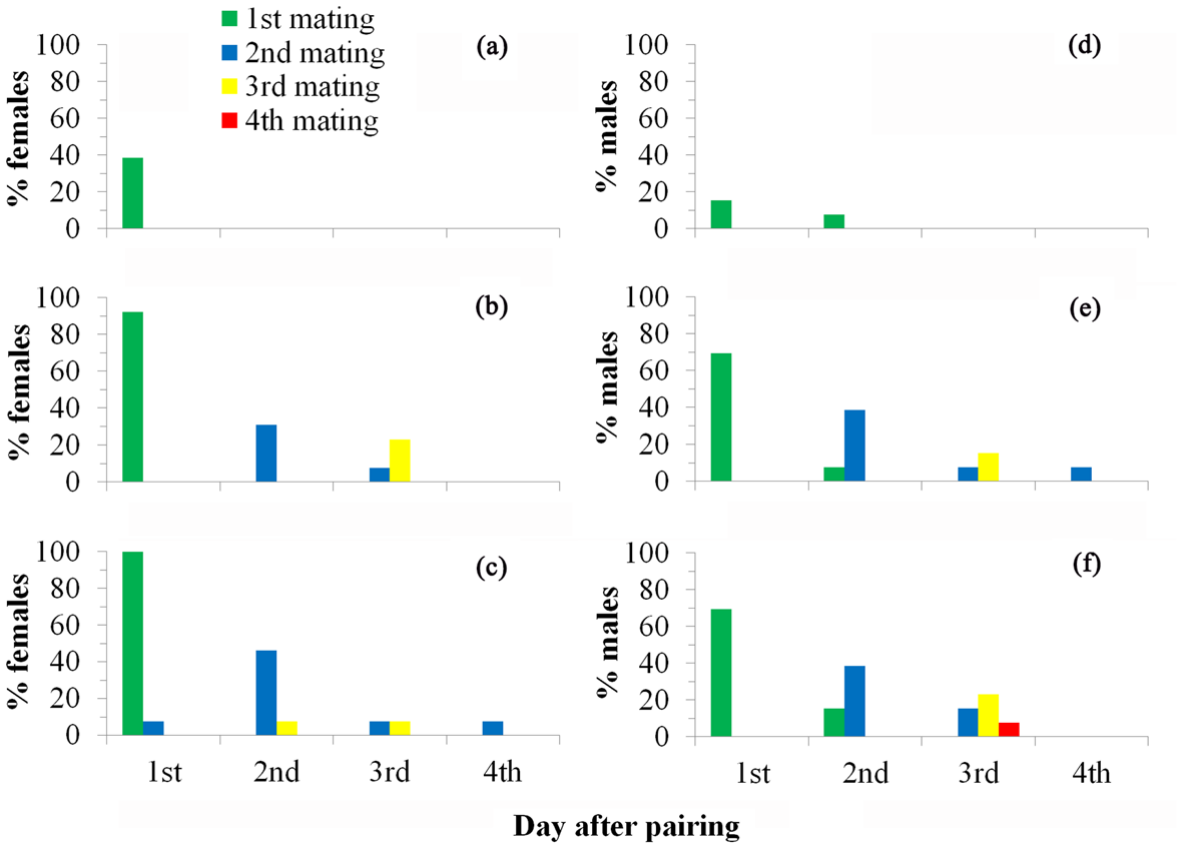
Effects of NTL RNAi on male and female lifetime mating and remating patterns. (**a**) mating pattern of NTL dsRNA females; (**b**) mating pattern of EGFP dsRNA females; (**c**) mating pattern of Water females; (**d**) mating pattern of NTL dsRNA males; (**e**) mating pattern of EGFP dsRNA males; (**f**) mating pattern of Water males.

**Figure 6:**
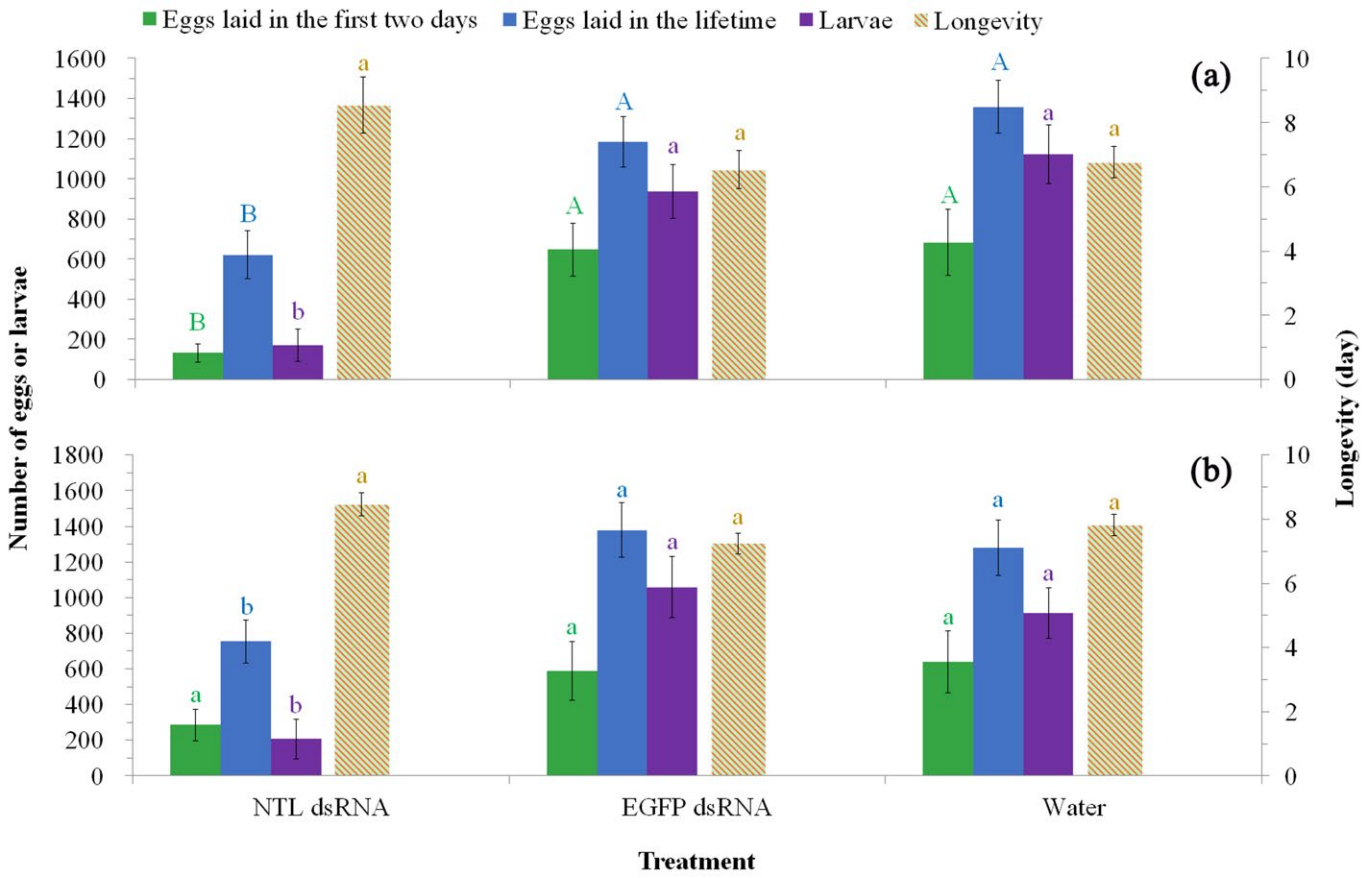
Effects of NTL RNAi on female (**a**) and male (**b**) reproductive output and longevity (mean number of days the adults live, i.e., the duration from moth eclosion to death). For each parameter (Eggs laid in the first two days, Eggs laid in the lifetime, Larvae or Longevity) of RNAi on female (**a**) or RNAi on male (**b**), bars with different letters are significantly different (different uppercase letters indicate *P* < 0.01 and different lowercase letters indicate *P* < 0.05); i.e., letters with the same color can be compared to derive the significance of difference between treatments in the same subgraph, (**a**) or (**b**).

However, NTL dsRNA females (feeding on *E. coli* expressing NTL dsRNA) showed significant lower calling rate (*χ*^2^ = 20.29, *P* < 0.0001 for the first scotophase and *χ*^*2*^ = 6.68, *P* < 0.05 for the second scotophase; Fig. 4a,c) and shorter calling duration (Table 3), and lower mating rate with wild males (*χ*^*2*^ = 14.20, *P* < 0.001 for the first scotophase and *χ*^*2*^ = 19.43, *P* < 0.0001 for the second scotophase; Fig. 4b,d) in comparison with control females (feeding on sterile water or *E. coli* expressing EGFP dsRNA). The lifetime mating events in these females were also recorded and presented in Fig. 5a–c. Only 38% NTL dsRNA females mated in their lifetime and no remating occurred in these females (mean mating rate is 0.38 time per female) while almost all control females mated at least once (92% for EGFP dsRNA females and 100% for Water females) and some of them mated up to four times (mean mating rate is 1.54 and 1.77 times per female for EGFP dsRNA and Water females, respectively). Fecundity data showed NTL dsRNA females laid significant fewer eggs in the first two days after pairing (*F*_2,36_ = 6.13, *P* < 0.01) and lifetime (*F*_2,36_ = 9.41, *P* < 0.001), and have fewer offspring (larvae) (*χ*^*2*^ = 18.53, *P* < 0.0001) than those of control females (Fig. 6a). NTL dsRNA females showed relative higher longevity than controls but not significantly different (*F*_2,36_ = 2.66, *P* > 0.05).

**Table 3:** Effects of NTL RNAi on female calling duration, number of male courtships and mating duration in *S. litura.*

Pairing patterns	Parameters measured	Water (control)	GFP dsRNA (control)	NTL dsRNA	*χ*^2^-value	*P *value
Treated female paired with untreated male	Female calling duration in the 1st scotophase (min)	68.5 ± 24.4a	55.4 ± 23.1a	7.69 ± 4.55b	3.53	0.039
	Female calling duration in the 2nd scotophase (min)	79.2 ± 27.4a	61.5 ± 24.3a	53.1 ± 20.9a	0.27	0.767
	Female mating duration (min)	43.3 ± 1.3a	43.5 ± 1.2a	42.0 ± 2.0a	0.20	0.823
Treated male paired with untreated female	Number of male courtships in the 1st scotophase	1.46 ± 0.33a	1.23 ± 0.23a	0.46 ± 0.27b	3.90	0.029
	Number of male courtships in the 2nd scotophase	1.46 ± 0.43ab	2.62 ± 0.56a	0.77 ± 0.34b	4.67	0.015
	Male mating duration (min)	44.7 ± 1.7a	44.0 ± 1.6a	43.3 ± 3.3a	0.07	0.930

Thirteen pairs were used for each Pairing pattern. Female calling durations or the numbers of male courtships were the means of all tested females or males in the 1st or 2nd scotophase. Female or male mating durations were the means of male or female matings occurred in the first two scotophases. Data were not normally distributed even after transformation and thus were analysed using the nonparametric Kruskal–Wallis test followed by Dunn’s procedure for multiple comparisons. For each parameter (in each line), values with different letters are significantly different (*P* < 0.05).

Similarly, NTL dsRNA males showed significant lower courting rate (*χ*^*2*^ = 19.57, *P* < 0.0001 for the first scotophase and *χ*^*2*^ = 12.55, *P* < 0.01 for the second scotophase; Fig. 4e,g) and fewer courtships (Table 3), and lower mating rate with wild females (*χ*^*2*^ = 14.92, *P* < 0.001 for the first scotophase and *χ*^*2*^ = 12.24, *P* < 0.01 for the second scotophase; Fig. 4f,h) in comparison with control males. The lifetime mating events in these males were also recorded and presented in Fig. 5d–f. NTL dsRNA males showed lower mating rate in their lifetime (23% mated) and no remating occurred in these males (mean mating rate is 0.23 time per female) while most control males mated at least once (77% for EGFP dsRNA males and 85% for Water males) and some of them mated up to three times (mean mating rate is 1.54 and 1.62 times per male for EGFP dsRNA and Water males, respectively). Fecundity test showed that wild females mated with dsNTL males laid fewer eggs in the first two days after pairing (but not significant: *F*_2,36_ = 1.70, *P* > 0.05) and lifetime (significant: *F*_2,36_ = 5.42, *P* < 0.01), and have significant fewer offspring (larvae) (*F*_2,36_ = 12.59, *P* < 0.01) than those of wild females mated with control males (Fig. 6b). NTL dsRNA males showed relative higher longevity than controls but not significant (*F*_2,36_ = 3.21, *P* > 0.05).

## Discussion

To date, NTL orthologs have been found in more than twenty arthropod species (Fig. 2). Most of these orthologs belong to Insecta species (Fig. 2) and more importantly, NTL has been proven to play an important function in the reproduction in three insect species, *D. melanogaster*, *T. castaneum* and *B. dorsalis*^7,8^. This evidence suggests that NTL is pervasive and may play essential roles in insect reproduction process.

The common cutworm moth *S. litura* is one of the key agricultural pests and is also notorious for developing insecticide resistance^30,31^. Developing environmental friendly control methods such as RNAi mediated management technique is imperative to control this pest. In the present study, we for the first time cloned and sequenced the NTL transcript in *S. litura*. This transcript contains a 1317 nucleotides ORF encoding a 438-amino acid protein (Fig. 1a). Twelve putative *Sl*-NTL neuropeptides were defined by flanking dibasic cleavage sites (combinations of K and R). Moreover, an amidated C-terminus was predicted for each mature peptide by a canonical amidation site (G). A complex enzymatic process is involved during the release of neuropeptides from their precursor proteins^45,46^. The C-terminal motifs of *Sl*-NTL is FWxxRamide, which is consistent to other species from Lepidoptera, Coleoptera, Orthoptera, Hemiptera and other non-insect species from Crustacea (Fig. 2). However, the C-terminal motifs of NTL is FxPxRamide in Diptera and in mites might be FxxxRamide. In addition, as showed in *S. litura* (Fig. 1) and other insects (Dataset S1), not all predicted peptides have the “Ramide” C-terminal structure, which can varied in the same or different species^7,8^. These variances on the C-terminal structure may reflect an evolutionary process based on the ligand–receptor activities^7,47^.

Real-time fluorescence quantitative analysis also showed that *Sl*-NTL expressed in both male and female adults’ heads, thoraxes and reproductive systems and showed variances. Tissue specific expression levels of NTL have been shown in other insects and the highest transcript levels of NTL were found in the CNS in *T. castaneum* and *B. dorsalis*^7,8^. The expression pattern of *Sl*-NTL in different developmental stages from larvae to adults (Fig. 3a) is quite similar to that of *B. dorsalis*^8^, i.e., lower expression levels during the larval stage, which increased to a higher level during the pupal stage, and then peaked at the beginning of the adult stage. *S. litura* adults eclose at dusk and no mating occurs in the eclosion scotophase (0-d-old). Maximum mating (approximately 70%) occurs during the subsequent scotophase after eclosion (1-d-old)^48^. The remaining unmated moths mate during the third scotophase (2-d-old). The correlation between NTL expression and adult sexual maturation in this species suggests this peptide has an important function in reproduction. However, the expression of NTL in *S. litura* is much lower during the egg stage while in *B. dorsalis* it is much higher (even higher than adult stage)^8^*.* This may suggest that NTL might have different functions during different developmental stage in different species.

To shed some light on the function of NTL and explore its potential as a target for novel insecticides, we knockdown NTL expression in both sexes by using bacteria-mediated RNAi and tested whether and how silencing of NTL will affect the developmental and reproductive fitness in this pest. Relative expression levels of *Sl-NTL* in NTL dsRNA males and females reduced (reduction percentage ranged from 45.88 to 82.70%) significantly in comparison with controls (Table 1). Both RNAi^49^ and bacteria intaking^50^ are likely to induce insect immune responses and thus may bring side effects on gene function study using bacteria-mediated RNAi. In the present study, knock down of NTL expression did not significantly affect the development, survival and morphology in *S. litura*. Moreover, our previous study also showed that feeding bacteria did not significantly affect the reproductive behavior and fecundity in *S. litura* (J. Xu, unpl. data). These results suggest that bacteria-mediated RNAi can be used for reproduction-related gene function analysis in this insect. Other studies^25,51^ also suggested that the bacteria-mediated RNAi can be a reliable technique for gene function study as these bacteria are non-pathogenic and the same and similar bacteria species are widely found in the gut of insects^53^. In the present study, NTL knock down significantly reduced adults’ reproductive behavior, including male courtship and female calling behavior in the first night (Fig. 4a,f), mating (reduced more than 50%) and remating pattern and rate (remating rate reduced more than 75%; no remating occurred in NTL knock down individuals while wild ones can mate up to 4 times) in both sexes in their lifetime (Figs. 4, 5). In *D. melanogaster*^7^ and *B. dorsalis*^8^, knocking down the expression of NTL also significantly reduced mating rate by about 40–75%. Similar to *T. castaneum*^7^, fecundity test also demonstrated that NTL knock down significantly reduced male and female productive output in *S. litura* (offspring gain reduced more than 70%; Fig. 6).

Based on the above results and similar results on NTL in other insect species^7,8^, we suggest that NTL may also play important roles in the process of reproduction in *S. litura*. However, the exact mechanism of NTL for influencing mating behaviors is still unclear. Previous studies have demonstrated that NTLs may regulate reproductive behaviors via activating their receptors (NTLRs; G protein-coupled receptors, GPCR) in insect based on ligand-receptor interaction analysis and NTLR-RNAi^7,8,47^. NTLR was expressed dominantly in the CNS in comparison with other tissues and NTLR mRNA was also confirmed in the digestive (midgut and hindgut) and reproductive systems^7,47^. These evidences suggested that NTL was released into the periphery, where it may bind to the receptor and implement various functions through GPCR signal systems. However, more investigation is required to reveal the signal cascades of NTLR in regulation reproductive behavior. Moreover, two NTLRs (Bm A32 and A33) were found in *B. mori*, which is different to other insect species that have only one copy of the NTLR^7^. And interestingly, Bm A32 was specific to BmNTL1, 3, and 5, which have the C-terminal FxxxRa consensus sequence, whereas BmNTLR A33 was specific to BmNTL10 and BmNTL11, which have the YxxxRa consensus sequence^7^. These results have suggested that the mechanism of NTL for influencing mating behaviors may be more complicated and varied in different insect taxa. Studies on the Sex Peptide (SP) and its receptor (SPR; also a GPCR) for the function on reproductive behavior in *Drosophila* have shown that the regulation mechanism is complicated, which is related to neural, physiological and molecular processes^52–54^. Therefore, future studies on NTL regulation mechanisms by taking account these (neural, physiological and molecular) processes are likely to achieve better progress in this field. The bacterial-mediated RNAi targeting NTL developed in this study have built a foundation for a better understanding on how the neuropeptide NTL is involved in reproductive physiology and may be a potential technique for pest control.

## Supplementary information

Supplementary information.

## Data Availability

All data generated or analysed during this study are included in this article and its Supplementary Information files.
