# Supplementary material for: Bacterial-mediated RNAi and functional analysis of Natalisin in a moth
Source: Sci Rep. 2021 Feb 25;11:4662. doi: 10.1038/s41598-021-84104-0 (PMC7907129; doi:10.1038/s41598-021-84104-0)
Supplement: Supplementary file 1 — Supplementary information. [file 41598_2021_84104_MOESM1_ESM.docx]

**Bacterial-mediated RNAi and Functional Analysis of *Natalisin* in a Moth**

Xia-Fei Wang^1,2,#^, Zhe Chen^1,#^, Xu-Bo Wang^1^, Jin Xu^1,^*, Peng Chen^3,^*, Hui Ye^2^

^1^Yunnan Academy of Biodiversity, Southwest Forestry University, Kunming 650224, China

^2^School of Life Sciences, Yunnan University, Kunming 650091, China

^3^Yunnan Academy of Forestry and Grassland, Kunming 650201, China

*Correspondence: Jin Xu, e-mail: xujin2798@126.com; Peng Chen, e-mail: pengchenn@126.com

^#^These authors contributed equally to this paper.

**Dataset S1** The Natalisin pre-propeptides and mature peptides. Signal peptide is in italics and underlined. Canonical amidation with di-basic signals is marked by bold and underlined fonts. The putative mature peptides were marked by blue fonts. Predicted mature peptides are aligned and presented under each pre-propeptide. Lower case “a” at the end of C-termini refers to amidation. Dots in the N-termini refer to uncertain cleavage sites. These sequences were collected and analysed base on Jiang et al. ([2013](#_ENREF_4)).

**Lepidoptera**

>*Spodoptera litura* Natalisin MK673156 [Lepidoptera; Glossata; Ditrysia; Noctuoidea; Noctuidae; Amphipyrinae; Spodoptera]

*MRRKVQTFLLFILIITTDVVLG*KANKTKTYKNHNKKQNSLKDKSENERV**KR**SLNDDDDRPFWPNK**GKK**LIGNDPNYNSKYDNDFAGNQKFAKSNDQVMKEEPFWGNR**GRR**EEEPFWGNR**GRR**DEIEPFWGNR**GRR**EIDEPFWGNR**GRR**ETDDPFWGNR**GRR**ETDDPFWGNR**GRR**DEEPFWGNR**GRR**KTSEPNWVRKQDLKESILNAINDVEEDIENLSRL**KK**SNADPNSFWTGR**GR**ENKLSMLFSGPLKNRANLPKAARLHGQTTEPGTVLDNRMYVDEPNYILVERT**GR**SSAEADDPYYISR**GKK**YYINYNLEQAARD**RR**GAIEEIVKSVRNDPYYIAR**GKK**DINLAKNGTTLHKEEYTKAKELICAAIDLIMIKNDKGKVKREIDDNDRDRRTIL**KK**LAAQLQMDPYFVSR**GKK**NQISGDKDNLQDFISNVADKCN

Sl01

Sl02

Sl03

Sl04

Sl05

Sl06

Sl07

Sl08

Sl09

Sl10

Sl11

Sl12

SLNDDDDRPFWPNK

..SNDQVMKEEPFWGNR

EEEPFWGNR

DEIEPFWGNR

EIDEPFWGNR

ETDDPFWGNR

ETDDPFWGNR

DEEPFWGNR

SNADPNSFWTGR

SSAEADDPYYISR

GAIEEIVKSVRNDPYYIAR

LAAQLQMDPYFVSR

>AXY04277.1 natalisin 2, partial [*Spodoptera exigua*] [Lepidoptera; Glossata; Ditrysia; Noctuoidea; Noctuidae; Amphipyrinae; Spodoptera] ([Llopis-Gimenez et al. 2019](#_ENREF_5))

*MRRKVQTFLLFILIITTDVVLG*KANKTKTYKNYHKKLNSLKDKSESERV**KR**SLNDDDDRPFWPNR**GKK**QIIGNDPNNYNSKYDSDFGSNQKFAKSNDQVMKEQPFWGNR**GRR**DSSMENLYGYTVDFPDYFVKHCEHCTEFAQKPNDYYGNIKD**RR**EDFVSPFWGNR**GRR**NSLESEGSEEDLFWGSR**GRR**QDQEPFWGNR**GRR**TENEPFWGNR**GRR**EEEPFWGNR**GRR**DEIEPFWGNR**GRR**EIDEPFWGNR**GRR**ETDDPFWGNG**GRR**EIEE

SLNDDDDRPFWPNR

..EQPFWGNR

EDFVSPFWGNR

NSLESEGSEEDLFWGSR

QDQEPFWGNR

TENEPFWGNR

EEEPFWGNR

DEIEPFWGNR

EIDEPFWGNR

Se01

Se02

Se03

Se04

Se05

Se06

Se07

Se08

Se09

Se10

ETDDPFWGNG

>XP_028170126.1 repetin-like [*Ostrinia furnacalis*] [Lepidoptera; Glossata; Ditrysia; Pyraloidea; Crambidae; Pyraustinae; Ostrinia]

*MHTRMYIFLLLLLLLSTDIALG*SVNKTKTTKISHKHNSAKYHLKAHSDRS**KR**SIDDDDENPFWPNR**GKK**ELNALINSEKESPFWATR**GRR**ESSSVENYIDAQKPYKSYLSKNDRYCENCFPSSNSDEVYGNVKHTRDDVTSPFWGNR**GRR**SSEELIDNFLPESLFWGSRSARQENEPFWGNR**GRR**QDDPPFWGHR**GRR**EDEPFWGNR**GRR**EETPFWGHR**GRR**DDVDDPFWGNR**GRR**DDFEPFWGNR**GRR**KDSEPFWSSR**GRR**KEDLKDSILHAINNVENDIENLSRL**KK**SDSEHNSFWGTRPRDSKLKHLFDGPRNRPHALTTEPRTVHDNRIYAEEPHYIIVERNSRSSAEDDPFYISR**GKK**YYINLPKAARD**RR**GAIEEIVKSVRNDPFYIAR**GKK**DDIVKVANTTMSHEEFVKAKELICATVELIMMKNENNKTKRDITDSERDRRTIL**KK**LAAQLQMDPYFVSR**GKK**CGDSNNDDNLVEFINEVAGKCN

SIDDDDENPFWPNR

ELNALINSEKESPFWATR

..HTRDDVTSPFWGNR

..QENEPFWGNR

QDDPPFWGHR

EDEPFWGNR

EETPFWGHR

DDVDDPFWGNR

DDFEPFWGNR

KDSEPFWSSR

SSAEDDPFYISR

GAIEEIVKSVRNDPFYIAR

LAAQLQMDPYFVSR

Of01

Of02

Of03

Of04

Of05

Of06

Of07

Of08

Of09

Of10

Of11

Of12

Of13

>ALM30330.1 natalisin precursor [*Chilo suppressalis*] [Lepidoptera; Glossata; Ditrysia; Pyraloidea; Crambidae; Crambinae; Chilo]

*MRRKMYIFLLVNLVLSTDLALG*SPNNKKFGNKIPQNKHVSKDETKNERS**KR**ALLEEEEHPFWPNR**GKK**ELINSDSENEQFWATR**GRR**ESSEENYMEDKTYTPQK**GR**LCKNCYPIGNSDAIYGNMKHTRDDTSPFWGMR**GRR**DSDEVEEDLFWGSR**GRR**HEVEPFWGNR**GRR**YEDTPFWGNR**GRR**EEEPFWGNR**GRR**EESPFWGNR**GRR**ENDEPFWGNR**GRR**EEPVVGPGNRVRQEDYEPFWGNR**GRR**KESESFWSSR**GRR**KQDLKEAILNAISVVENDIENLSRLRRNSDDQDTFLDNKSKE**RR**LQHLVNDPLRTHQSFLKTNEDPGTLLDNTMYVEQPHYVVVERSSRSSAEDDPFFISR**GKK**YFLNFDLEKAVRD**RR**GAIEEIVKSVRNDPFYIAR**GKK**DAEFDKKGNSTKAMSTLSREEFLKARELICSTIDLIANKNGKNKRNTSLQKLAAQLQMDPYYVSRGKKTDTTNSDSLEDFINEVAAKCN

ALLEEEEHPFWPNR

ELINSDSENEQFWATR

.. HTRDDTSPFWGMR

DSDEVEEDLFWGSR

HEVEPFWGNR

YEDTPFWGNR

Cs01

Cs02

Cs03

Cs04

Cs05

Cs06

Cs07

EEEPFWGNR

EESPFWGNR

ENDEPFWGNR

EEPVVGPGNRVRQEDYEPFWGNR

KESESFWSSR

SSAEDDPFFISR

GAIEEIVKSVRNDPFYIAR

Cs08

Cs09

Cs10

Cs11

Cs12

Cs13

>*Bombyx mori* PROTEIN:BGIBMGA010189-PA [Lepidoptera; Glossata; Ditrysia; Bombycoidea; Bombycidae; Bombycinae; Bombyx] ([Jiang et al. 2013](#_ENREF_4))

*MKNKTIQLIVLFLILITSDLTFG*KGNYNKKNLRTHQQNKKHNNTKTKFESERIKRSTNDFGTVQFWTNKEIVRPKYFNSEDSLQNIEDAPIY**GK**IHNEPPFWAIR**GRR**DSSSSEIYTEPHRLESDRHDLQQNYDHVTKIGLWNEPDLKHPANFWANR**GRR**NPLDFDRSFMLEPLWERDLRQENDPFWGNR**GKK**EEAFWSSK**GK**TEENPFWANR**GRR**LDQLEDRTNRDSNTDVDPFWGSR**GRR**KDMEFIKDHKKENLKQSILNAINDVEKDVKTLSRL**RR**SPGAGLNFWLNR**GR**DSKLKHLFNRNSNNKAYVSNAGSSTVNTVNPSNAETLSDSRIYADEPHYILLERNSRSSAEDDPFYISR**GKK**YYLKYNFGRP**GR**DRRGAIEEIVKSVRNDPYYIAR**GKK**DLTNESSTQRNQYSKIKELVCTAIDMVLKKNNGDVVKRDITDSERDRRTIL**KK**LAAQLQNDPYFASR**GKK**SSADEGNHLNEFIDSIAEKCS

IHNEPPFWAIRa

..IGLWNEPDLKHPANFWANRa

..DLRQENDPFWGNRa

EEAFWSSKa

TEENPFWANRa

..DSNTDVDPFWGSRa

SPGAGLNFWLNRa

..SSAEDDPFYISRa

YYLKYNFGRPa

..SVRNDPYYIARa

LAAQLQNDPYFASRa

Bm01

Bm02

Bm03

Bm04

Bm05

Bm06

Bm07

Bm08

Bm09

Bm10

Bm11

>*Manduca sexta* Msex005511-PC [Lepidoptera; Glossata; Ditrysia; Bombycoidea; Sphingidae; Sphinginae; Sphingini; Manduca]

*MSSKLTASALLLLILSIAFVYS*KDVNKTKLFNKKHTNLKPTNHDRTKRSSNGNEASTVWLNKGLQIVPESSGYNGNIQDKITSNLNTF**R**QLDIEPFWATR**GRR**DSSSTEDYNDSEYSGTVNKLSAVVCNGCPTPSHLNYRNVKIS**R**DEYSPFWGNR**GRR**GSYENNDPAEVFWGSR**GRR**EDNEPFWGNR**GRR**KDDPPFWGNR**GRR**EEEPFWGNR**GRR**QSAAWSHNS**R**YEDEPFWGNR**GRR**EDPEPFWGNR**GRR**EDTEPFWGNR**GRR**EDSEPFWGNR**GRR**KDAEPFWGTR**GRR**KEDLKESILTAIDNVESDIENLSRL**RR**SDDMNSFWANR**GR**DSTLKFLPSAPVKSKTLLAPNSETVHDSRIYAEQPKFILVERNS**R**SSAEEDPYFISR**GKK**YYLNYNLIKAARD**RR**GAIEEIVKSVRNDPYYIAR**GKK**RREDARVNNATKHEEMQKIKDLVCSAIDLITINTPNDKVKREINENERDRRTIL**KK**LAAQLQIDPYFVSR**GKK**NEENGDKDYLYAFINDVAEKCDY

Ms01

..QLDIEPFWATRa

..DEYSPFWGNRa

GSYENNDPAEVFWGSRa

EDNEPFWGNRa

DDPPFWGNRa

EEEPFWGNRa

..YEDEPFWGNRa

EDPEPFWGNRa

EDTEPFWGNRa

EDSEPFWGNRa

DAEPFWGTRa

SDDMNSFWANRa

..SSAEEDPYFISRa

GAIEEIVKSVRNDPYYIARa

LAAQLQIDPYFVSRa

Ms02

Ms03

Ms04

Ms05

Ms06

Ms07

Ms08

Ms09

Ms10

Ms11

Ms12

Ms13

Ms14

MS15

>*Danaus plexippus* gi|357623042|gb|EHJ74348.1| hypothetical protein KGM_04896 [Lepidoptera; Glossata; Ditrysia; Papilionoidea; Nymphalidae; Danainae; Danaini; Danaina; Danaus]

*MGLKTNLGTSIVPQKVTAT*VHTNEYLKIKFKIMQKHIVILLIFFLTSQYIVYGINENKTKTVKLLNKKTNNYEQKSKQERTKRSLNEGSVQYYLPDEDTFLDKVDWYENNNDES**GKR**LLNKETPFWATR**GRR**DSASEENYADLPLTTHIYYNFLGKTKNVHKLPAYLM**K**QRHDEIDAPFWGNR**GRR**ASEESNENENNLFWANR**GRR**QDDDPFWGTR**GRR**QEEAPFWGNR**GRR**ENLYSTFKGDDIEPFWGNR**GRR**ETQNQDLDAN**GKTRIRSLALLNEETEPFWGNRGRR**KLDVKESIVKAISEIEKNIDRLNKDRNSKIKSLLNGPNKNPVGSIHDITKLKPNNEIRSKPFGQNTIHDNRIYAEEPHYILVERS**GR**SSEEDDPFFITR**GKK**YNCLQLDKAR**GRR**GALEDIF**KSVRNDPYYIARGKK**DTTPHFENSSSLQKKMLKAKDLICATIELTKANKVKRQASDSERDRRTTL**KK**LALQLQMDPYFVSR**GKK**FDSNVDLNSVEELINQISVTCN

LLNKETPFWATRa

..QRHDEIDAPFWGNRa

ASEESNENENNLFWANRa

QDDDPFWGTRa

QEEAPFWGNRa

..GDDIEPFWGNRa

TRIRSLALLNEETEPFWGNRa

SSEEDDPFFITRa

YNCLQLDKARa

..SVRNDPYYIARa

LALQLQMDPYFVSRa

Dp01

Dp02

Dp03

Dp04

Dp05

Dp06

Dp07

Dp08

Dp09

Dp10

Dp11

**Diptera**

>*Aedes aegypti* gi|157135410|ref|XP_001656645.1| hypothetical protein AaeL_AAEL003260 [Diptera; Nematocera; Culicoidea; Culicidae; Culicinae; Aedini; Aedes; Stegomyia]

*MKTTISVGICVLCCLVALIAA*EAFGYQTNRDKHSTAETAEVSKNEQNSVKPADHGSRSTNSGEHVLEYVPGDSSQVNEHQHE**KR**EILTHPSGGDIPLLDTQQHLR**GRR**LPFYVDEPRYVVI**GR**PSLFQSNGGFSFIPQR**GRK**SDPPQPSSISATALE**KR**ELSIQQMLQGSDYFVPNR**GKK**RLSSGIS**KK**IKFDDILGSDELFIPNR**GKK**ELFDLFPAMTR**GR**MLNDFQPYLSKKNVLNTIQNVGS**K**ESDSGGELFYPTR**GKK**NILENLAQSQDTFFSSRGKRNPLLLAEDEGLSPWEYLAGADDDSSYDADLPPLDSYQSNKIIT

Aa01

EILTHPSGGDIPLLDTQQHLRa

PSLFQSNGGFSFIPQRa

ELSIQQMLQGSDYFVPNRa

IKFDDILGSDELFIPNRa

ELFDLFPAMTRa

ESDSGGELFYPTRa

Aa02

Aa03

Aa04

Aa05

Aa06

>*Culex quinquefasciatus* gi|170029723|ref|XP_001842741.1| conserved hypothetical protein [Diptera; Nematocera; Culicoidea; Culicidae; Culicinae; Culicini; Culex; Culex]

*MSRTTSLGMCVLCCLVVALIVEA*LEFDQQQQQSAKHSRPPATSATVKNDPKMLEYVPEGIPADMAPQDSEHHRQHDKRDAMP**RR**PSLEDLLAQQWPEPPNLR**GRR**LPLYAEEPRFILLRQPTQQQQHHSSPSFSFIPQR**GR**KSDPLLSTLAKRELSIKQMLEGGDYFVPNR**GKK**ATGVGISKKVKFDDILGSDELFIPNR**GKK**ELFELFGGGGRAINELQPYYYNKKNVLTAIQNVG**GK**NDGTAEELFYPTR**GKK**ANVLDNLAQNADTFFSSR**GKR**IPVAWNLLNQNTNGNDDDPWEYLTAATADDSSSYDADFALDRYQTNKIIA

PSLEDLLAQQWPEPPNLRa

..QPTQQQQHHSSPSFSFIPQRa

..QMLEGGDYFVPNRa

..FDDILGSDELFIPNRa

NDGTAEELFYPTRa

ANVLDNLAQNADTFFSSRa

Cq01

Cq02

Cq03

Cq04

Cq05

Cq06

>*Anopheles gambiae* str. PEST gi|158286987|ref|XP_560286.3| AGAP005277-PA [Diptera; Nematocera; Culicoidea; Culicidae; Anophelinae; Anopheles] ([Riehle et al. 2002](#_ENREF_7))

*MALKRPRISVGVMAVCCGVLS*LSVVARESASPSEAETGGRKYVAVHHQPVHPGSFSSPAAMEWMPVTTPLTPAPKPIDEERHRKTREALQFSRPALPSDGSITTSPTPSQIIRRRRMEPKIRGLPSPLVFMPAR**GRR**FGSFEIPAEKRQITIEQMLE**K**GDYFVPNR**GKK**APTNGELIKK**GK**FDVLLGGSPDEYFFPNR**GKK**QYLLAYDAGSNIRPLVTNTARGDTVPLDDKVSSFAVPSLQHLHQPLVNRL**RR**NLLENLANEHKDTFFSSR**GKR**IVPMAQELMFLPPKALSDDQQDDAVPEGLTDARAQDFTGQANDLDALFWNEDLLLPLEQAGS

..GLPSPLVFMPARa

GDYFVPNRa

FDVLLGGSPDEYFFPNRa

NLLENLANEHKDTFFSSRa

Ag01

Ag02

Ag03

Ag04

>*Mayetiola destructor* maker-A1.29-snap-gene-11.52-mRNA-1 (A1.29:1090304..1095747) [Diptera; Nematocera; Sciaroidea; Cecidomyiidae; Mayetiola]

MATQPTINNNQTETDMKSIVKLNSMQHDFLKQIPRMAEITLPTNFIDWDTKQLNTPSDVHSTNHALISAPESLDDPFIPQR**GRR**QSNSQTYDDPFIPMR**GRR**SKAQPWDDPFIPMR**GRR**AEKAAWDDPFYPMR**GRR**AEKSAWDDPFFPMR**GRR**ADQNGWDDPFFPMR**GRR**DDRFDDPFIPMR**GRR**SISKSSDMKMRQRRELFDQPFSAIYTPTEEVIVKLNGVYDTIHSNQLKHASNSNEWLNEAPTKSQPMQLIESMNLPLLSRGYLPGNKRSASKKKLQQQQSNQFFIPNNFVSNPDDDGDNSLMNDYSFNNYPESPQMSRAHIMHKNRIQRPIHLLMKSSTDLSQNRDAANSARGRDTSFLF

Md01

..ISAPESLDDPFIPQRa

QSNSQTYDDPFIPMRa

SKAQPWDDPFIPMRa

AEKAAWDDPFYPMRa

AEKSAWDDPFFPMRa

ADQNGWDDPFFPMRa

DDRFDDPFIPMRa

Md02

Md03

Md04

Md05

Md06

Md07

>AQM36728.1 natalisin [*Bactrocera dorsalis*][Diptera; Brachycera; Muscomorpha; Tephritoidea; Tephritidae; Bactrocera; Bactrocera] ([Gui et al. 2017](#_ENREF_1))

*MKITLPVLVATLTIYCGATVL*SLPPSLIATAASLARHHVRQTRDSRGSATITADVNVNAHAFAQNDGGGGIYDSMDNINDDGAVDNVYSDADAVAEMSYEEDEGNNASNDILMLLSGEQDVMKFLSWAMQQLYPYQHFPQLNGSEGAEYYYPGMFNWKKLNLSGHLEPPLVVEEPHYVIVRREQLEDDLPPGIRIED**RK**PKQRSALFQSIFNLNHGPQYKDDPFIPPR**GRK**HNLPDLDALLNRYETFVPNR**GKR**DKIKDIFKYDDLFFPNR**GKK**QGPPKAATVVQSGDDRANEVNTVEIAPEDNVDGGGQTDGRRGLLLRDQVDRSDDSNLTVSPAAVAISRLLAGWMKCLQPHELCVPLKRVVDVTRDGNVSGTQRSGFEKNNDDNSNNYSDTIDSDIAVADGYNSGNGGRPDRVARFRRVPTTFGGITRIAANRLRSMPTTIKSAERQQNKSPTRRQGRLPHQQPAMLRYFIRQLRQKQAQRRGSWQWPLLANQVRTNINDVDLLTWRQQQQDNMNKPLAAEHSPLQQEQIEQPKGQQTEHHHLQHLFGGVSGITAGRSSPETADIGGI

PKQRSALFQSIFNLNHGPQYKDDPFIPPR

HNLPDLDALLNRYETFVPNR

DKIKDIFKYDDLFFPNR

Bd01

Bd02

Bd03

>*Drosophila melanogaster* gi|281361759|ref|NP_001163608.1| CG34388, isoform D [Diptera; Brachycera; Muscomorpha; Ephydroidea; Drosophilidae; Drosophila; Sophophora] ([Jiang et al. 2013](#_ENREF_4))

*MRLTLAWLSLCLAIYCGGGHG*HGNVVLSLPPSLIATATKAALSHQRQQKQQQQHQKKDARVLFDSPADALRDMMHNGNGNGNGPMDSGKFSLSDVEQPAAQRSEDFNRNAYDLGARQSAPQEIAMGMELGMGLGLGPNNYRTTPPHRYWGQRCQGRSGGSGTSKCPQEYYRTMLAARNKEALSRLHMQLSSMQDSDSGASSSSDSEEEHVDDEEQSNNEVFMLLTGEQDLMKFLHWAMQVLYPIERPLGNLSDGAAENYYPGMFLWKKLNLSGHLEPPLIVDEPQYVLV**RR**EKLFDGYQFGEDMSKENDPFIPPR**GRK**HSGSLDLDALMNRYEPFVPNR**GKR**DKVKDLFKYDDLFYPHR**GKK**HRNLFQVDDPFFATR**GKK**LQLRDLYNADDPFVPNR**GKR**HLTASAGKLGETMAGGGKWPDDSNNYWPLRMSTHKINGYDQSVRPSLSVEDAAASLASWRLPANRLHSTRSMSADLRQQLLLPHVRFIGNPNMRQQQQQQQQHQVKTSSWQAEERLRRSILAPGESNDAHETQLTLSHPANPHLVTDTDNLNI

EKLFDGYQFGEDMSKENDPFIPPRa

HSGSLDLDALMNRYEPFVPNRa

DKVKDLFKYDDLFYPHRa

Dm01

Dm02

Dm03

Dm04

HRNLFQVDDPFFATRa

LQLRDLYNADDPFVPNRa

Dm05

**Orthoptera**

>AKN21249.1 natalisin precursor, partial [*Locusta migratoria*] **[**Orthoptera; Caelifera; Acrididea; Acridomorpha; Acridoidea; Acrididae; Oedipodinae] ([Hou et al. 2015](#_ENREF_2))

*MPHAAAWTWLAAIATLALA*YQEGENATLLEEGAGGRVKRAGAPELGLADDGRSRRQEPLYVEEPAWVLLD**RR**ETGEPLQQVSAAAAAAMPAELVDPFWVAR**GRR**REISSSSASAEEPFWAAR**GRR**EMELFWPAR**GKR**HQQEEVSGTAEDAEEEAGLSGQWSAVRERRAGLRDYLASLSAS**RR**ASAGGQYFVPAR**GKR**DGESKRAAGSAR**RR**MTLGSGSAEEPFWAAR**GRR**GEAARRG

..MPAELVDPFWVAR

REISSSSASAEEPFWAAR

EMELFWPAR

ASAGGQYFVPAR

MTLGSGSAEEPFWAAR

Lm01

Lm02

Lm03

Lm04

Lm05

**Coleoptera**

>*Tribolium castaneum* Chromosome X new prediction [Coleoptera; Polyphaga; Cucujiformia; Tenebrionidae; Tenebrionidae incertae sedis; Tribolium] ([Jiang et al. 2013](#_ENREF_4))

MHVSIKWLLVLTLTGVHAEEPRKRNDRFSFEEPFILEQNDDDVSCSIGNCV**KR**ASGQEEFGPFWANR**GKK**DPTYTRSKLFAEEPHWILV**RR**DDNDINDNEPFYVTR**GKK**NSDEERKLFWKNLIKKRSFVQGLGDLQYFTNGIKHDRNERNVDFP

Tc201 ASGQEEFGPFWANRa

Tc202 DDNDINDNEPFYVTRa

**Hemiptera**

>*Aphis gossypii* cDNA Trans (-3) gi|289077628|gb|GW501819.1|GW501819 IvAg3p_08135 [Hemiptera; Sternorrhyncha; Aphidomorpha; Aphidoidea; Aphididae; Aphidini; Aphis; Aphis]

LMDARTKLYLIGVAYFVQLINGDARIS**R**ADISAVLGENDDPNFWPSR**GRR**NSPEPKFKQYLERKYNMNTLNGHLEKPLYVDEPMWLTID**RR**AEGDDDYFWVTR**GRR**GNSWKHPTASRLSTNSNYRYRDESDNKIK

ADISAVLGENDDPNFWPSRa

AEGDDDYFWVTRa

Apg01

Apg02

>*Acyrthosiphon pisum* gi|328711852|ref|XP_003244659.1| LOC100571085 [Hemiptera; Sternorrhyncha; Aphidomorpha; Aphidoidea; Aphididae; Macrosiphini; Acyrthosiphon]

*MELMDARTKLYLISVAYFVQLINGD*ARIS**R**ADISAVLGENDDPNFWPSR**GRR**NSPEPKFKQYLERKYNMNTLNGNLQKPLYVDEPMWLTID**RR**AEGDDDYFWVTR**GRR**GNSWKHPTSARLSTNSNYNYRDEPDNKIK

..ADISAVLGENDDPNFWPSRa

AEGDDDYFWVTRa

Ap01

Ap02

>*Nilaparvata lugens* cDNA Trans (-3) gi|331992489|gb|HS460043.1|HS460043 BL-42209 [Hemiptera; Auchenorrhyncha; Fulgoroidea; Delphacidae; Delphacinae; Nilaparvata]

ELQSLLQERGVGSDEEPPFWANR**GR**SLPCDGIGQDGALFAEEPYWVMMD

Nl01 ..GVGSDEEPPFWANRa

>*Rhodnius prolixus* RPTMP02028-RA [Hemiptera; Heteroptera; Panheteroptera; Cimicomorpha; Reduviidae; Triatominae; Rhodnius] ([Ons et al. 2016](#_ENREF_6))

*MNPVHTLLVALISVGLQETSS*VGEKCDKAVCGEERRSDV**R**AVLGSSEAEPGFWPTR**GRR**GDSSSTEEVQPPFWAHR**GR**EERPCDSSSVNSLNHLYAQEPKFLLIH**RR**DTMEQDPFWVSR**GR**KRSQKSNFAYLEETSLTAGEGGGELITVTNSFAGEMRGLWSLGRLPVQVKSHLNIQYKWHLSLANITNTQPEQVP

..AVLGSSEAEPGFWPTRa

GDSSSTEEVQPPFWAHRa

DTMEQDPFWVSRa

Rho01

Rho02

Rho03

**Arachnida**

>uncharacterized protein LOC107369011 [*Tetranychus urticae*] XP_015792419.1 [Arachnida; Acari; Acariformes; Trombidiformes; Prostigmata; Eleutherengona; Raphignathae; Tetranychoidea; Tetranychidae; Tetranychus]

*MAFVRCSIFILLLGIWPLVWA*SDSVEPSFDHQLNSRESKDGKERIDGLSYFLDNQRSLIPLVSSPSSSSLSSSSSPLTPSSPSSASSVATLLRNSNNNNLYDLYGTKGNSYLATSADENEKNFADDYVVDLP**GKR**RFIPLR**GRK**DDLFFHPRSDSFYTAFDGYR**KK**AAFTGMR**GKK**MDLSSPVNNRQGALIRNMMNRNGYGAMDDSPLISFANLNDVGAY**KR**SRPFAAMR**GKR**SIQTSNLLPF

RFIPLRa

AAFTGMRa

SRPFAAMRa

Tu101

Tu102

Tu103

>uncharacterized protein LOC107368892 [*Tetranychus urticae*] XP_015792263.1 [Arachnida; Acari; Acariformes; Trombidiformes; Prostigmata; Eleutherengona; Raphignathae; Tetranychoidea; Tetranychidae; Tetranychus]

*MLLKNGSFLFLSLLFLCVTWNSVSG*SDNIDSPFNQQILDSHEIKSNEIGKHYATSSLSYFLDNQRSIIPLSTSPKFNKKNNIYSNNVKENNFYDLYGIRESTYSPENGNNLIDLPGKRKFIQLDQHLNDWIR**RR**SAFNGMR**GKK**VNLPLPSSPYTLRNSFGIYHWNRTNDNLIV**KR**ARPFAAML**GKR**YALRRGRARFYASRG

Tu201 SAFNGMRa

Tu202 ARPFAAMLa

>*Varroa destructor* Vdes_2.0_scaffolds.fa (scf7180002156110.. scf7180002174645) [Arachnida; Acari; Parasitiformes; Mesostigmata; Gamasina; Dermanyssoidea; Varroidae; Varroa] ([Jiang et al. 2016](#_ENREF_3))

MYTPMCQGCLSRSAHRMSMHAHTEQPHYNEELEQTKLLNILSRIFPSSQQARYLYDDLLYGGA**KR**SIGGPAPGFVGAR**GKK**GGVPGFVGAR**GKR**FWIGSTEERRR

Vd01 SIGGPAPGFVGAR

Vd02 GGVPGFVGAR

**Crustacea**

>AWK57549.1 natalisin/WXXXRamide, partial [*Cherax quadricarinatus*] [Crustacea; Multicrustacea; Malacostraca; Eumalacostraca; Eucarida; Decapoda; Pleocyemata; Astacidea; Parastacoidea; Parastacidae; Cherax] ([Tuan Viet et al. 2016](#_ENREF_8))

WIAR**GKK**VVVHPFWVAR**GKK**EVNPFWVTR**GK**EEAAARPFWIAR**GKK**EVHPFWVAR**GKK**EEESHPFWVAR**GKK**GETNPFW

VVVHPFWVAR

EVNPFWVTR

EEAAARPFWIAR

EVHPFWVAR

EEESHPFWVAR

Cq01

Cq02

Cq03

Cq04

Cq05

>*Procambarus clarkia* [Crustacea; Malacostraca; Eumalacostraca; Eucarida; Decapoda; Pleocyemata; Astacidea; Astacoidea; Cambaridae; Procambarus] ([Veenstra 2015](#_ENREF_9))

*MIRRSDVMGCLWVATCLASALA*ITKPVEDDSQEKQVLPGEEASSYVTSRLLPSYTSDSSGFWPLR**GKR**PPSGDLTTA**GRR**TLVMGSDGAFWLTR**GKK**AYQGHQPFYWGSNQDLWGETPYTRKPGEGAEEVTSLRPPSQFPTPGDTKQSSRGKRDSGGPFWVSR**GKK**DGGPFWVAR**GKR**VMGTDLAWGANPWIMGMRSQDVDQDGVISYLSSDDIFFSKREGGGPFWTSR**GKK**PAGGGPGGSPPLWVSRV**GK**SSGDKTFWVAR**GKK**DAPHTHPFWISR**GKR**DDANITSFWVAR**GKK**AGESHPFWIAR**GK**ADHATHPFWE**RR**GKDEPEGHPLWITR**GKK**EVKTKPFWVSREESDGAPYWVSGGRREDQRPSDGGGVPVPRTVARDSHEDPVMAYLLQVLAEGQNDTVPEDARSKTWL

..LLPSYTSDSSGFWPLR

TLVMGSDGAFWLTR

GKRDSGGPFWVSR

DGGPFWVAR

.. EGGGPFWTSR

SSGDKTFWVAR

DAPHTHPFWISR

DDANITSFWVAR

AGESHPFWIAR

GKDEPEGHPLWITR

Pc01

Pc02

Pc03

Pc04

Pc05

Pc06

Pc07

Pc08

Pc00

Pc10

>*Caligus clemensi* cDNA Trans (+2) gi|226260388|gb|GO410317.1|GO410317 EST_ccle_evs_1030648 [Crustacea; Maxillopoda; Copepoda; Siphonostomatoida; Caligidae; Caligus]

GEIAISIQLPWHSELIGILP**GK**TMKIGPTWAWLWTLFLFHQCIGISGDDTSLPLKSSSDFWATR**GRR**SGEENIQSPEDLIFWANR**GKR**VNYNLPRPNGFLFPTSLSGPSSNSILDKRKYFNLPRPNGFMFPSSSSVGSNGKRSDFYPYNDDAAANDEFMFKKRNNIHLPKPNNFFFLSSKNAQSPGGKRGDETFKNYNLPNPNGFLFPSKQGKRASKLNWNMVTPNGFFFPAAAKAQFSKREAILEDLLARLYS**KR**GEEVTGDIFFVGR**GKR**ADVDMDTFFCGR**GRR**SDQG

..DDTSLPLKSSSDFWATRa

SGEENIQSPEDLIFWANRa

GEEVTGDIFFVGRa

ADVDMDTFFCGRa

CC01

CC02

CC03

CC04

>*Daphnia pulex* gi|321475615|gb|EFX86577.1| hypothetical protein DAPPUDRAFT_97675 [Crustacea; Branchiopoda; Diplostraca; Cladocera; Anomopoda; Daphniidae; Daphnia]

*MELIKIIFVLASGWATLAIA*GNTDQDMFWAAR**GKK**ASIEGNWPDSVVMEPFVAAVYD**KR**DGTFWAAR**GKK**YAADGGDGVPFWATR**GKK**GDLEIPFWAAR**GKR**IPQSEMNETEEEGNRREKRSAGRDTSIIHSGQRFRNRPA**R**PASQAAEPFWAAR**GKK**NSNVSKFPLL

Dpu01

GNTDQDMFWAARa

DGTFWAARa

YAADGGDGVPFWATRa

GDLEIPFWAARa

..PASQAAEPFWAARa

Dpu02

Dpu03

Dpu04

Dpu05

**References:**

Gui S. H., H. B. Jiang, X. Q. Liu, L. Xu, and J. J. Wang. 2017. Molecular characterizations of natalisin and its roles in modulating mating in the oriental fruit fly, *Bactrocera dorsalis* (Hendel). Insect. Mol. Biol., 26: 103-112.

Hou L., F. Jiang, P. Yang, X. Wang, and L. Kang. 2015. Molecular characterization and expression profiles of neuropeptide precursors in the migratory locust. Insect. Biochem. Molec, 63: 63-71.

Jiang H., D. Kim, S. Dobesh, J. D. Evans, R. J. Nachman, K. Kaczmarek, J. Zabrocki, and Y. Park. 2016. Ligand selectivity in tachykinin and natalisin neuropeptidergic systems of the honey bee parasitic mite *Varroa destructor*. Sci. Rep., 6: 19547.

Jiang H. B., A. Lkhagva, I. Daubnerova, H. S. Chae, L. Simo, S. H. Jung, Y. K. Yoon, N. R. Lee, J. Y. Seong, D. Zitnan, et al. 2013. Natalisin, a tachykinin-like signaling system, regulates sexual activity and fecundity in insects. P. Natl. Acad. Sci. USA., 110: E3526-E3534.

Llopis-Gimenez A., Y. Han, Y. Kim, V. I. D. Ros, and S. Herrero. 2019. Identification and expression analysis of the *Spodoptera exigua* neuropeptidome under different physiological conditions. Insect. Mol. Biol., 28: 161-175.

Ons S., A. Lavore, M. Sterkel, J. Pedro Wulff, I. Sierra, J. Martinez-Barnetche, M. Henry Rodriguez, and R. Rivera-Pomar. 2016. Identification of G protein coupled receptors for opsines and neurohormones in *Rhodnius prolixus*. Genomic and transcriptomic analysis. Insect. Biochem. Molec, 69: 34-50.

Riehle M. A., S. F. Garczynski, J. W. Crim, C. A. Hill, and M. R. Brown. 2002. Neuropeptides and peptide hormones in *Anopheles gambiae*. Science (Washington D C), 298: 172-175.

Tuan Viet N., S. F. Cummins, A. Elizur, and T. Ventura. 2016. Transcriptomic characterization and curation of candidate neuropeptides regulating reproduction in the eyestalk ganglia of the Australian crayfish, *Cherax quadricarinatus*. Sci. Rep., 6: 38658-Article No.: 38658.

Veenstra J. A. 2015. The power of next-generation sequencing as illustrated by the neuropeptidome of the crayfish *Procambarus clarkii*. Gen. Comp. Endocr., 224: 84-95.
